# The Effect of Disease and Season to Hepatopancreas and Intestinal Mycobiota of *Litopenaeus vannamei*

**DOI:** 10.3389/fmicb.2019.00889

**Published:** 2019-04-24

**Authors:** Juan Li, Haiying Jiang, Linmiao Li, Xiujuan Zhang, Jinping Chen

**Affiliations:** Guangdong Key Laboratory of Animal Conservation and Resource Utilization, Guangdong Public Laboratory of Wild Animal Conservation and Utilization, Guangdong Institute of Applied Biological Resources, Guangzhou, China

**Keywords:** intestine, ITS amplicon, hepatopancreas, *Litopenaeus vannamei*, mycobiota

## Abstract

Increasing evidence has manifested that the gut bacterial microbiota of shrimps is closely related to the environmental factors, host developmental stage and health status like that of humans and animals does. These studies have provided an important guidance for improving shrimp culture benefits. In practice, aside from bacteria, eukaryotic microorganisms dominated by fungal microbiota (mycobiota), also play a key role in host growth, metabolism and homeostasis. However, little so far is known about the mycobiota in the digestive tract of shrimp. In this study, we used high-throughput sequencing of internal transcribed spacer 1 region to characterize the hepatopancreas and intestinal mycobiota of Pacific white shrimp and their connections with disease incidence and seasonal variation. The results showed that the hepatopancreas and intestinal mycobiota of *Litopenaeus vannamei* are dominated by the phyla Ascomycota and Basidiomycota, and the genera *Alternaria*, *Tuber*, *Hortaea*, *Sarocladium*, and *Stagonospora*. The fungal microbiota significantly varies under the influence of disease and seasonal variation. Sick shrimps had a higher level of potential pathogenic fungus, *Candida* in the intestine. Healthy shrimps had a higher abundance of the genera *Didymella* and *Filobasidium* in the gut, and *Pyrenochaetopsis* in the hepatopancreas. Of note, most of the fungi carried by Pacific white shrimps were pathogens to humans. This study has revealed the intestinal and hepatopancreas mycobiota of *L. vannamei* and the effects of diseases and seasonal variation to the mycobiota. Our study provides important guidance for Pacific white shrimp farming and sheds further insight on the fungal microbiota.

## Introduction

The digestive system hosts a large number of microorganisms dominated by bacteria, which constitute an enormous microbial ecosystem called the microbiota (O’Hara and Shanahan, 2006; Clemente et al., 2012). The microbiota has multiple functions, such as the maintenance of the functional stability and metabolic balance in the digestive system (Xue et al., 2015; Sommer et al., 2016; Sonnenburg and Backhed, 2016) and the regulation of the host immune response (Hooper et al., 2012; Palm et al., 2015). Therefore, it is critical for the host. Although the bacterial microbiota is dominant, eukaryotic microorganisms also occur (Neville et al., 2015; Sam et al., 2017). Fungi are the paramount eukaryotic microbiota (Neville et al., 2015). Recently, many studies showed that the gut fungal population was tightly correlated to host immune- and metabolic-modulatory pathways (Mar Rodriguez et al., 2015; McAleer et al., 2016; Huseyin et al., 2017; Sokol et al., 2017) and are thus receiving an increasing amount of attention.

Crustaceans are the second most species-rich subphylum in Arthropod. Of these, shrimps have attracted extensive attention because of their commercial importance. It has been reported that shrimp farming can reach 3 million tons of production each year (Zhang et al., 2016). Pacific white shrimp, *Litopenaeus vannamei*, is the most popular shrimp species, contributing to their survival advantages, such as fast growth rate and strong adaptability to environment. However, high frequency of diseases has severely influenced the shrimp farming. Recent studies revealed that the intestinal bacterial microbiota of the Pacific white shrimp is closely related to host age, health conditions and immune response (Xiong et al., 2015; Cornejo-Granados et al., 2017). Stable enteric bacterial microbiota enhances the immunity of the shrimp and their resistance to external pathogens (Rungrassamee et al., 2016). Based on the results, a novel farming approach that aggregates the shrimp tolerance to diseases by maintaining or reestablishing a “healthy” gut microbiota has been developed and brought deep and positive effects on shrimp farming (Xiong, 2018). Besides, a study of pacific white shrimps has disclosed that the gut bacterial community regularly varies over shrimp disease progression and developmental stage (Xiong et al., 2017). The new findings led to that a more accurate earlier diagnosis approach that monitors microbiota has appeared and has gradually been applied to shrimp farming (Xiong et al., 2017; Xiong, 2018).

Of note, a recent study targeted the 18S rRNA gene to uncover that the gut eukaryotic microbiota of cohabitating shrimps affects the digestion and nutrition absorption and consequently alter the growth performance (Dai et al., 2017). However, the fungal community, as the most influential population in the gut eukaryotic microbiota, has not been clearly explored due to the insufficiency of sequencing depth in that study. In addition, a culture-based study found that there were various fungal pathogens in the body of the Pacific white shrimp (da Silva et al., 2011; Karthikeyan et al., 2015), which may influence the health status of the farmed shrimp. However, the number of fungi that can be cultured were limited (Huffnagle and Noverr, 2013), and the interspecies ecological interaction that existed inside the digestive tract has also barely been detected based on the conventional culture-dependent method.

In addition, differing from that of vertebrates, the shrimp digestive system is continuous and is comprised of the stomach, hepatopancreas and intestine. Of these, the hepatopancreas plays an important role in regulating host innate immunity, and it is also a vital organ, which is responsible for digestion and absorption (Cornejo-Granados et al., 2017). However, to date, few studies have focused on the bacterial microbiota in the other digestive organs apart from the intestine, not to mention the fungal microbiota. Interestingly, several recent studies of the shrimp gut bacteria found that aside from the intestine, the hepatopancreas is also a habitat of many bacteria, which can respond to the variation of environment and health condition and is closely related to the host diet (Cheung et al., 2015; Cornejo-Granados et al., 2017). However, the variation of the mycobiota in the different parts of shrimp digestive tract, such as hepatopancreas and intestine, remains unrevealed.

In this study, we used high-throughput sequencing technology to analyze the hepatopancreas and intestinal fungal microbiota of *L. vannamei*. The aim was to understand: (1) the composition of the hepatopancreas and enteric fungal microbiota, (2) the seasonal effects on the composition of the hepatopancreas and enteric mycobiota, and (3) the effects of disease on the composition of the hepatopancreas and enteric mycobiota of *L. vannamei*.

## Materials and Methods

### Ethics Statement

All experimental animal protocols in this study were approved by the committee on the Ethics of Animal Experiments of the Guangdong Institute of Applied Biological Resources and followed the basic principles.

### Sampling

In summer, according to the disease status, three representative infected pools and a pool that cultured healthy shrimps were chosen. In winter, we randomly chose four pools for sampling as no diseased shrimps have been found. All of the culture pools chosen in this study have used the same artificial food (HAID GROUP, China). A total of 50 white shrimps from eight farms were sampled, as shown in Table 1. To avoid contamination, the live shrimp that was fished with a screen was immediately placed in 50 mL sterilized collection tubes. The collection tubes were put into ice to quickly bring back to the local lab. Each shrimp was thoroughly washed with sterile water and placed in a sterile plate. The washed samples were placed on the sterile workstation for biopsies to collect the hepatopancreas and intestinal tissue. The tissue removed was immediately placed in 2 mL EP tubes and quickly placed in dry ice. The samples were stored at −80°C in the lab until the nucleic acids were extracted.

**Table 1:** Shrimp samples used to detect hepatopancreas and intestinal fungi.

Sample time	Farming pool	Number	Tissue (group name)	Disease state
2017/6	SA	6	Intestine (SAC), hepatopancreas (SAG)	White feces
	SB	5	Intestine (SBC), hepatopancreas (SBG)	Black gill
	SC	12	Intestine (SCC), hepatopancreas (SCG)	Retarded growth
	SD	3	Intestine (SDC), hepatopancreas (SDG)	Healthy
2017/12	WA	5	Intestine (WAC), hepatopancreas (WAG)	Healthy
	WB	6	Intestine (WBC), hepatopancreas (WBG)	Healthy
	WC	7	Intestine (WCC), hepatopancreas (WCG)	Healthy
	WD	6	Intestine (WDC), hepatopancreas (WDG)	Healthy

### DNA Extraction, PCR Amplification, and Sequencing

For each sample, the total genomic DNA was extracted using a PowerSoil^®^ DNA Isolation Kit (MO BIO, United States) according to the manufacturer’s instructions. The DNA isolation was quantified using a ND-2000C spectrophotometer (NanoDrop, United States). Internal transcribed spacer 1 region (ITS1) was amplified using the universal primer set ITS1F 5′-CTTGGTCATTTAGAGGAAGTAA-3′ and ITS2 5′-GCTGCGTTCTTCATCGATGC-3′ (Mukherjee et al., 2014; Li et al., 2017). PCR reactions were performed using Phusion^®^ Q5 High-Fidelity DNA Polymerase (New England Biolabs, United Kingdom) under the program [95°C for 5 min, 15 × (95°C for 30 s, 50°C for 30 s, 72°C for 40 s), 72°C for 7 min, hold at 4°C; 98°C for 30 min, 15 × (98°C for 30 s, 65°C for 30 s, 72°C for 30 s), 72°C for 5 min, hold at 4°C]. Sterilized water was used as the negative control. The primer set attaching barcode sequences was used during the second PCR amplification. After agarose gel electrophoresis, the PCR products was purified by using VAHTSTM DNA Clean Beads (Vazyme, China), followed by Solexa PCR, second bead purification. The final products were pooled to generate the sequencing libraries. The libraries were sequenced on an Illumina HiSeq platform (Illumina Hiseq 2500) using HiSeq Rapid Kit V2 (500 cycle) (Illumina, United States) following the manufacturer’s instructions.

### Data Processing

Based on the unique barcodes, we assigned the sequence to each sample before removing the barcode and primer sequence using QIIME v1.8.0 (Caporaso et al., 2010). FLASH v1.2.7 was used to perform merging of the PE reads to obtain merged sequences (raw tags) (Magoc and Salzberg, 2011). The minimum length of the overlap was set to 10 bp, and the maximum mismatch ratio in the overlap area was set to 0.2 (Magoc and Salzberg, 2011). Trimmomatic v 0.33 was applied to conduct a comparatively stringent quality control to generate a set of high quality clean tags (Bolger et al., 2014), followed by removing the chimeric sequences using UCHIME v 4.2 (Edgar et al., 2011) and the non-fungal sequences with ITSx v 1.1.1 (Bengtsson-Palme et al., 2013). Later, the high-quality effective tags were clustered to generate operational taxonomic units (OTUs) using UCLUST v 1.2.22 at 97% similarity (Edgar, 2013). Low-abundant OTUs (the number of sequence was below three) were filtered, and normalized OTUs were generated (Bokulich et al., 2013). According to the UNITE database v 7.2^1^ (Koljalg et al., 2013), the representative sequence of each OTU was annotated with RDP Classifier v 2.2 (Wang et al., 2007).

### Statistical Analysis

The normalized OTUs were used to plot the species distribution bar charts at each taxonomic level, followed by the construction of phylogenetic trees using ClustalW2^2^.

The abundance data were used to calculate the α diversity (Chao 1, ACE, Richness, Shannon–Wiener and Simpson diversity indices) using Mothur v.1.3.0^3^ (Schloss et al., 2009). The differences among the groups were compared using a one-way analysis of variance (ANOVA). We plotted the rarefaction curve and the species relative abundance curve to evaluate the sufficiency of the sequencing depth (Wang et al., 2012; Koljalg et al., 2013).

The β diversity of each sample group was calculated based on the Bray–Curtis distance. Principal coordinate analysis was completed with the R package “ape,” and a clustering dendrogram was constructed using an unweighted pair-group method with arithmetic mean (UPGMA). The differences of β diversity between the groups were detected with a permutational multivariate analysis of variance (PERMANOVA).

Linear discriminative analysis effect size (LEfSe) analysis was performed at each taxonomic level to screen for the molecular markers (Biomarkers) of each group (Segata et al., 2011).

### Sequence Data Accession Number

The sequencing data generated from the one hundred samples described in this study are available in a sequence read archive (SRA) at the NCBI under the accession number PRJNA495902.

## Results

### Assessment of Sequence Data

One hundred samples were sequenced on the Illumina Hiseq 2500 platform to generate 12.56 Gb dataset and 9,501,484 pairs of reads. Of those, 8,960,483 tags passed the strict quality control and were processed into 8,735,797 high-quality chimera-free effective tags (87,358 on average) (Supplementary Table S1). A total of 9,804 OTUs were generated at 97% similarity (Supplementary Table S2). Subsequently, the OTUs, that were below three in the sum of the sequence number, were screened to obtain 8,491 effective OTUs (Supplementary Figure S1). The diversity indices were calculated based on the OTU data. The rarefaction curves and the species relative abundance curve reached a plateau, reflecting the sufficiency the sequencing depth (Supplementary Figures S2, S3).

### General Pattern of the Gut and Hepatopancreas Mycobiota of *Litopenaeus vannamei*

**FIGURE 1:**
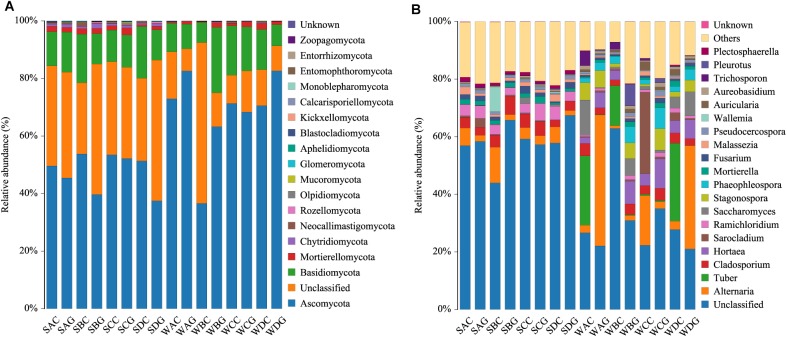
Species distribution of each group in 50 shrimps. Panel **(A)** at the phylum level; panel **(B)** at the genus level (top 20=). The sample ID referred to Table 1.

A total of 17 phyla, 53 classes, 127 orders, 325 families, 734 genera, and 990 species were identified from the classifiable sequences. As shown in Figure 1A, Ascomycota was the most dominant phylum and occupied 65.32% of the total fungal community, followed by Basidiomycota (12.45%). Other phyla in low abundance were, in descending order, Mortierellomycota (1.19%), Chytridiomycota (0.21%), and Neocallimastigomycota (0.19%).

At the genus level, the top 20 members were affiliated with *Alternaria* (12.83%), *Tuber* (5.97%), *Hortaea* (4.93%), *Sarocladium* (3.95%), *Stagonospora* (3.47%), *Saccharomyces* (3.43%), *Cladosporium* (3.28%), *Phaeophleospora* (3.08%), *Ramichloridium* (1.37%), *Mortierella* (1.13%), *Fusarium* (1.05%), *Auricularia* (1.00%), *Pleurotus* (0.94%), *Polyporus* (0.93%), *Pseudocercospora* (0.86%), *Trichosporon* (0.84%), *Aureobasidium* (0.84%), *Curvularia* (0.74%), *Malassezia* (0.69%), and *Candida* (0.59%) (Figure 1B).

### Comparison Between the Gut and Hepatopancreas Mycobiota

**FIGURE 2:**
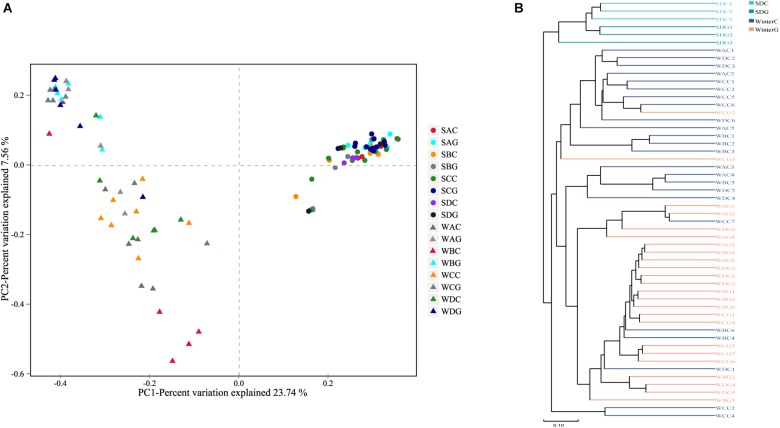
Boxplots depict differences of beta diversity between intestinal and hepatopancreas samples. **(A)** Summer shrimps; **(B)** winter shrimps. Beta diversity of intestinal and hepatopancreas mycobiota. **(A)** Principal coordinate analysis (PcoA) was based on Bray–Curtis distance. **(B)** A clustering dendrogram was constructed using an unweighted pair-group method with arithmetic mean (UPGMA).

**FIGURE 3:**
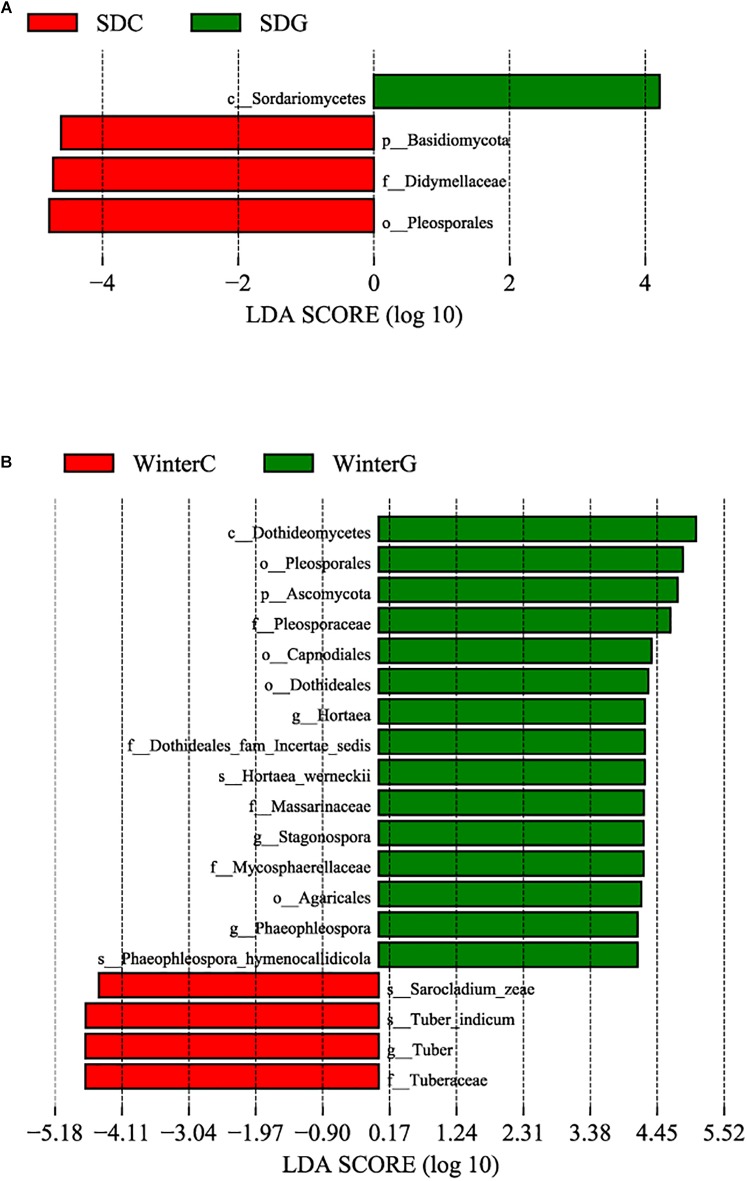
Differences among intestinal and hepatopancreas groups were determined by linear discriminative analysis effect size (LEfSe). The highlighted taxa were significantly enriched in the group that corresponds to each color. LDA scores can be interpreted as the degree of difference in relative abundance. **(A)** Comparison between the summer healthy intestinal and hepatopancreas samples; **(B)** comparison between the winter intestinal and hepatopancreas samples. p_, phylum, c_class, o_order, f_, family, g_, genus and s_, species; WinterC, winter intestinal group; WinterG, winter hepatopancreas.

A comparison of the α diversity was conducted between the intestinal and hepatopancreatic fungal microbiota. The significance between the groups was analyzed using an ANOVA. To eliminate the interference of diseases, when analyzing the difference between the summer intestinal and hepatopancreas samples, we only chose the samples collected from pool SD (the intestinal group as SDC, the hepatopancreas group as SDG). The results showed that there were no detectable significant differences in the richness estimator and diversity estimator between the summer intestinal and hepatopancreas group (Supplementary Table S3A). For β diversity, although UPGMA cluster analysis seemed to show that the summer samples divided into two clades, the PCoA and PERMANOVA results both showed an overlap of the two group. This indicated the high similarity in community composition and diversity between the two groups (Figure 2 and Table 2). LEfSe analysis was used to capture the distinguished fungi between the groups. Basidiomycota, Pleosporales and Didymellacea showed a higher level in the intestinal group, and Sordariomycetes was significantly enriched in the hepatopancreas group. However, no typical representative fungi were found in genus and species level (Figure 3A).

**Table 2:** PERMANOVA results based on Bray–Curtis distance.

Comparision	Source of variation/ pairwise comparison	SS	*F*	*R*^2^	*P*	Significant
Intestine/hepatopancreas	SDC/SDG	0.040	1.388	0.258	0.100	
	WinterC/WinterG	0.087	16.987	0.270	0.001	^∗∗∗^
Summer/winter	SDC/WinterC	0.028	5.114	0.170	0.001	^∗∗∗^
	SDG/WinterG	0.105	11.801	0.321	0.001	^∗∗∗^
Diseased/healthy	SAC/SDC	0.010	4.315	0.381	0.015	^∗^
	SBC/SDC	0.007	1.734	0.224	0.136	
	SCC/SDC	0.004	1.007	0.071	0.319	
	SAG/SDG	0.013	2.819	0.287	0.032	^∗^
	SBG/SDG	0.002	0.209	0.340	0.793	
	SCG/SDG	0.012	3.043	0.190	0.049	^∗^

*^∗^P < 0.05, ^∗∗∗^P < 0.0001. WAC, WBC, WCC, WDC were merged into WinterC; WAG, WBG, WCG, WDG were merged into WinterG.*

**FIGURE 4:**
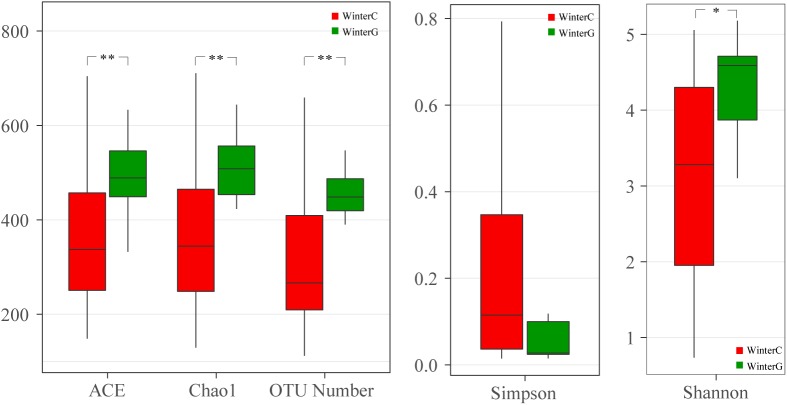
Boxplots depict differences of alpha diversity between winter intestinal and hepatopancreas samples. WinterC, winter intestinal samples; WinterG, winter hepatopancreas. ^∗^*P* < 0.05, ^∗∗^*P* < 0.01.

When comparing the winter intestinal group with the winter hepatopancreas group, the hepatopancreas group had a higher level of OTU number, ACE, Chao, and Shannon indices than the intestinal group; the trend was reversed in Simpson index (Figure 4 and Supplementary Table S3A). The β diversity of each group was analyzed based on a Bray–Curtis distance. PCoA results reflected that the intestinal and hepatopancreas group were divided into two sets (Figure 2). The UPGMA cluster analysis showed that despite disturbing by four winter intestinal samples, the hepatopancreas samples (with the exception of two samples from pool WC) together clustered into a separate branch (Figure 2). The significant difference between the intestinal and hepatopancreas group was confirmed by a PERMANOVA, as shown in Table 2. LEfSe analysis results showed that the hepatopancreas group was significantly enriched in the genera *Hortaea*, *Phaeophleospora*, and *Stagonospora*, while the intestinal group had a higher abundance of *Tuber* and *Sarocladium zeae* (Figure 3B).

### Comparison of the Mycobiota Between the Summer and Winter Samples

The influence of the season on the intestinal and hepatopancreas fungal microbiota was analyzed. To avoid the effects of diseases to the results of analysis, only SDC and SDG from summer have been used to perform the comparison analysis. The results demonstrated that there were no significant differences in the richness estimator and the community diversity estimator, as shown in Supplementary Table S3B.

To fully explore the differences in the community membership and diversity between the groups, we calculated the β diversity of each sample based on the Bray–Curtis distance. The PCoA results indicated that the summer and winter samples distinctly clustered into two sets (Figure 2A). The UPGMA cluster analysis also showed the same results (Figure 2B). The significant differences have been confirmed by a PERMANOVA as shown in Table 2.

**FIGURE 5:**
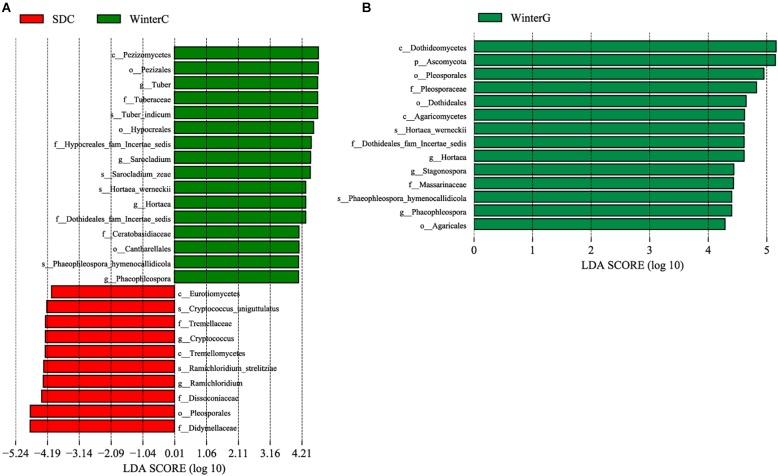
Differences among summer and winter groups were determined by LEfSe. The highlighted taxa were significantly enriched in the group that corresponds to each color. LDA scores can be interpreted as the degree of difference in relative abundance. **(A)** Comparison between summer and winter intestinal samples; **(B)** comparison between summer and winter hepatopancreas samples. p_, phylum, c_class, o_order, f_, family, g_, genus and s_, species; WinterC, winter intestinal group; WinterG, winter hepatopancreas.

Linear discriminative analysis effect size analysis was performed to screen the seasonally differential fungi in the intestine and hepatopancreas. For the shrimp intestine, three classes, four orders, seven families, six genera and six species presented a significant alteration in different groups (Figure 5A). The summer group had a significantly higher abundance of the genera *Cryptococcus* and *Ramichloridium*. The winter group presented a significant increase in the genera *Tuber*, *Sarocladium*, *Hortaea*, and *Phaeophleospora*. For the shrimp hepatopancreas, there were significant differences in two classes, three orders, three families, two genera and two species (Figure 5B). The winter hepatopancreas group showed an increased abundance of the genera *Hortaea*, *Phaeophleospora*, *Stagonospora*, but no typical representative fungi were found in the summer hepatopancreas group.

### Comparison of the Mycobiota Between Diseased and Healthy Groups

To detect the effects of disease on the fungal microbiota of *L. vannamei*, we compared sick intestinal groups (the intestinal samples from SA: SAC; the intestinal samples from SB: SBC, and the intestinal samples from SC: SCC) with healthy intestinal group (SDC), and sick hepatopancreas groups (the hepatopancreas samples from SA: SAG, the hepatopancreas samples from SB: SBG and the hepatopancreas samples from SC: SCG) with a healthy hepatopancreas group (SDG). The results showed that only the SAC group presented a significantly dissimilarity with the healthy intestinal group in OTU number and Shannon diversity. There were no detectable significant differences in α diversity between the rest of sick intestinal groups and healthy intestinal group, and between the sick hepatopancreas groups and the healthy hepatopancreas group (Supplementary Table S3C).

The differences in β diversity among the groups were compared using the PCoA and PERMANOVA (Figure 2 and Table 2). The PERMANOVA results showed that apart from the SAC group, there were no significant differences in community membership and community diversity between the sick and healthy intestinal groups. When the effects of disease on the hepatopancreas mycobiota were compared, the sick groups (SAG and SCG) showed a significant difference with the healthy group (SDG).

**FIGURE 6:**
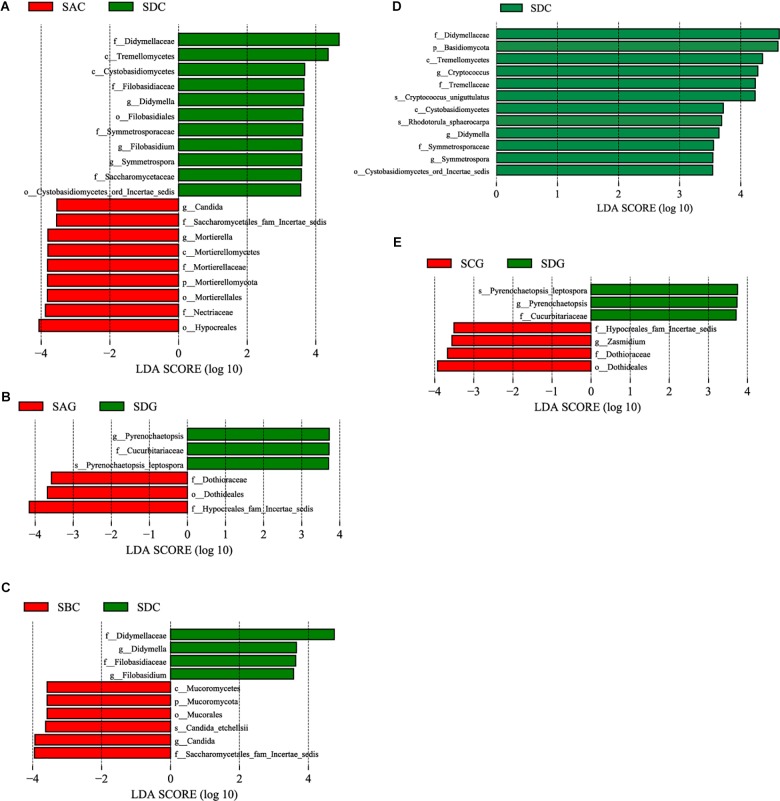
Differences among diseased and healthy groups were determined by LEfSe. The highlighted taxa were significantly enriched in the group that corresponds to each color. LDA scores can be interpreted as the degree of difference in relative abundance. **(A)** Comparison between SAC and SDC; **(B)** comparison between SAG and SDG; **(C)** comparison between SBC and SDC; **(D)** comparison between SCC and SDC; **(E)** comparison between SCG and SDG. p_, phylum, c_class, o_order, f_, family, g_, genus and s_, species; WinterC, winter intestinal group; WinterG, winter hepatopancreas.

Linear discriminative analysis effect size analysis was used to screen the representative fungi in each group. First, we compared the sick SA group with the healthy SD group (Figure 6A,B) and found that in the gut, the sick group (SAC) had a significantly higher abundance in the genera *Candida* and *Mortierella*. The healthy group (SDC) had a higher abundance in the genera *Didymella*, *Filobasidium*, and *Symmetrospora*. In the hepatopancreas, no genera showed a significantly increased abundance in the sick group (SAG), but the genus *Pyrenochaetopsis* was significantly enriched in the healthy group (SDG). The comparison between the SB group and the SD group (Figure 6C) showed that in the gut, the genus *Candida* was enriched in the sick group (SBC). In the healthy group (SDC), there was a significant increased abundance in the genera *Didymella* and *Filobasidium*. In the hepatopancreas, no representative fungi were found in the sick (SBG) and healthy (SDG) group. Finally, we analyzed the differential fungi between the sick SC group and the healthy SD group (Figure 6D,E). The results demonstrated that in the intestine, the genera *Cryptococcus*, *Didymella*, and *Symmertrospora* in the healthy group (SDC) significantly outnumbered those in the sick group (SCC). In the hepatopancreas, the number of the genus *Zasmidium* was larger in the sick group (SCG). The healthy group (SDG) had a higher level of the genus *Pyrenochaetopsis*.

## Discussion

### Composition Analysis of the Intestinal and Hepatopancreas Mycobiota of *L. vannamei*

Our study revealed the composition of the hepatopancreas and gut mycobiota in *L. vannamei* is highly diverse. *Alternaria*, *Tuber*, *Hortaea*, *Sarocladium*, and *Stagonospora* were identified as the most dominant five genera in the shrimp mycobiota (Figure 1B). Interestingly, *Candida* and *Saccharomyces*, which account for a large portion in humans and other animals (Neville et al., 2015; Hallen-Adams and Suhr, 2017; Schei et al., 2017; Li et al., 2018), were low-abundant in the shrimp intestinal and hepatopancreas mycobiota. It has been reported that *Candida* species had a higher level in hosts with carbohydrate diets (Hoffmann et al., 2013). Our recent study of the gut microbiota in bats with diverse diets has revealed that the number of *Candida* in the gut of insectivorous bats was significantly less than that of phytophagous bats (Li et al., 2018). In this study, the cohabitating Pacific white shrimp were fed with the same artificial food, which is high in protein and relatively low in carbohydrate. Thus, the low abundance of *Candida* species might partially be attributed to the specific diet. In addition, distinguishing from the bacterial microbiota, the fungal microbiota occurs primarily from the intake (De Schryver and Vadstein, 2014; Hallen-Adams and Suhr, 2017; Sam et al., 2017). *L. vannamei* is an aquatic anthropod. Many recent studies showed that environment factors have considerably influenced the shrimp gut bacterial microbiota by providing environment-associated microbes and disturbing the composition of the colonized microbial community (De Schryver and Vadstein, 2014; Chen et al., 2017; Cornejo-Granados et al., 2018; Huang et al., 2018; Xiong et al., 2018). Therefore, the aquatic environment may greatly affect the mycobiota of the shrimp, and thus shape the shrimp mycobiota that significantly differs from that of terrestrial organisms.

A previous culture-dependent study showed that the most primary fungal taxa in Pacific white shrimp were *Aspergillus*, *Penicillium*, and *Fusarium* (da Silva et al., 2011), and the presence of *Penicillium* was verified afterwards in another culture-based study (Laich and Andrade, 2016). Unexpectedly, in our study, *Penicillium* species were not found in the intestine or the hepatopancreas. This might contribute to the geographical divergence, which results in the specific aquatic microbiomes and environmental factors of each region (Seetharam et al., 2015; Chen et al., 2017).

### Comparison Between the Intestinal and Hepatopancreas Mycobiota

Many studies showed that as a result of the different niches and function of each part of the digestive system, the diversity and composition of the bacterial microbiota in different parts differed from one to another (Schoster et al., 2013; Zhao et al., 2015; He et al., 2018). Recent studies of the shrimp microbiota revealed that despite the lack of significant differences among the bacterial microbiota in each part of the intestine, the bacterial microbiota in the hepatopancreas was significantly distinguished from those in the intestine (Tzuc et al., 2014; Cornejo-Granados et al., 2017). However, little attention so far was paid to the eukaryotic microbial population in the shrimp digestive system, and none on the fungal population. In this study, our results revealed that for the summer shrimp, the fungal microbiota in the hepatopancreas was extremely homologous with that in the intestine (Figure 2A, Table 2, and Supplementary Table S3A). However, when compared to the mycobiota of the winter shrimp, the hepatopancreas group showed a higher richness and Shannon diversity than the intestinal group (Figure 4) and there were significant differences in the community membership and structure between the intestine and hepatopancreas (Figure 2 and Table 2). Previous studies confirmed that external fungi were a primary source of the gastrointestinal mycobiota (De Schryver and Vadstein, 2014; Hallen-Adams and Suhr, 2017). Like most of aquatic organisms (Deus and Petrere-Junior, 2003; Guilherme and Rosa, 2014), the shrimp dietary strategies seasonally vary. Attributed to the optimal temperature and higher metabolic rate in the summer, the summer shrimp eat faster and more than the winter ones. However, in the winter, the feeding rate significantly reduces, especially when the environmental temperature is below approximately 15°C, although the shrimp is able to survive at the lowest temperature of approximately 6°C. The reduction of the shrimp feeding frequency in the winter led to the decrease in the number of food-sourced fungi. Therefore, in winter, after the digestion treatments of the hepatopancreas, the fungi that have already been low in the number would be much fewer when arriving at the intestine.

### The Influence of Seasonal Variation on the Mycobiota of *L. vannamei*

The disease incidence of farmed aquatic animals presents a significantly seasonal alteration, which is higher in the summer but relatively lower in the winter. Recent studies showed that the gut microbiota is involved in regulating the immune system of the shrimp (Cornejo-Granados et al., 2017), and a stable microbiota can improve the host resistance to external pathogens (Xiong et al., 2015). In this study, although the comparison of alpha diversity indices showed there were no significant differences between the summer and the winter mycobiota, PCoA, UPGMA cluster analysis, and PERMANOVA demonstrated that the breeding season is a decisive factor shaping the shrimp mycobiota (Figure 2A,B and Table 2). It has been reported that the mycobiota is less stable than those of bacteria and is greatly determined by diet and fungi in the environment (Hallen-Adams and Suhr, 2017; Sam et al., 2017). Aquarium microbiome responses to the temperature variation (Van Bonn et al., 2015) and the high temperature of the summer can easily and quickly promote pathogen growth. Recent studies have demonstrated that shrimp gut microbiota is highly susceptible to environmental factors, even over short timescales (Cornejo-Granados et al., 2018; Huang et al., 2018; Xiong et al., 2018). The alteration of the number of the pathogens in the aquatic environment not only results in shrimp infections, but also directly changes the composition of the shrimp gut microbiota (Rungrassamee et al., 2016; Chen et al., 2017). Additionally, environment variation also indirectly interferes the stability of the microbiota (Huang et al., 2018; Xiong et al., 2018). Therefore, the intake of external pathogenic or opportunistically pathogenic fungi and the environmental changes might reduce the stability of the microbiome, especially the fungal population. The unstable mycobiota may negatively affect the immune function of the shrimp farmed in the summer and cause the increase of the disease incidence to some extent.

### The Influence of Disease on the Mycobiota of *L. vannamei*

The gastrointestinal microbiota (both bacteria and fungi) is sensitive and responds to the disease state of the host. In this study, the intestinal mycobiota of the sick group SA showed significant differences in OTU number and Shannon diversity compared with those of the healthy control, but no significant differences were found between the other sick groups and the healthy group (Supplementary Table S3C). Previous studies have shown that the diversity of the gut mycobiota in sick humans has a significant alteration compared with that of healthy humans. The variation of the diversity is inconsistent in patients suffering from different types of illnesses. There was an increased fungal diversity in the gut of Crohn’s disease patients (Li et al., 2014; Liguori et al., 2016) but a decrease in the alpha diversity in ulcerative colitis (Pineton de Chambrun et al., 2012) and inflammatory bowel disease patients (Sokol et al., 2017).

When compared to the effect of disease on the composition and structure of the mycobiota, apart from SAC, no significant differences were detected between the sick intestinal groups and healthy intestinal groups as well (Table 2). However, for the hepatopancreas mycobiota, there were significant differences between the two sick groups (SAG and SCG) and the healthy group (Table 2). The results indicated that suffering white feces significantly influenced the diversity, composition and structure of the gut mycobiota of the shrimps.

Of note, we found that pathogenic *Candida* (Banjara et al., 2016) in the intestine of the two sick groups (SAC and SBC) significantly outnumbered that of the healthy group (Figure 6A,C). Disease might alter the homeostasis among the autochthonous microbes. The alteration resulted in that the number of the pathogens which should have been a normal commensal increased. For the healthy group, the number of plant-pathogenic *Didymella* (Barilli et al., 2016; Ranjbar Sistani et al., 2017) and *Filobasidium*, presented a significant increase in the gut compared with the sick groups (Figure 6A,C,D). The healthy hepatopancreas group had a significantly higher abundance of *Pyrenochaetopsis* than the sick hepatopancreas groups (Figure 6B,E). Those fungi may be fungal indicators of the healthy shrimps.

### Fungal Pathogens From *L. vannamei*

Foodborne diseases are a hot topic around the world. Of these, food safety problems derived from microbial infections have become one of the problems of greatest concern (Liu et al., 2004; Xu and Zhang, 2012). Pacific white shrimp is a favored food commodity because of the delicious taste and rich nutrition but was also identified as a potential reservoir and disseminator of many human bacterial pathogens (Zhang et al., 2015). However, little is known about the fungal pathogens harbored by Pacific white shrimps.

Our results showed that *L. vannamei* harbored a variety of human fungal pathogens (Figure 1B and Supplementary Table S2). Of these, species from the three genera *Aspergillus*, *Candida*, and *Cryptococcus* are primarily responsible for the lethal fungal infections. *Hortaea werneckii* (Sharmin et al., 2002; Abliz et al., 2003) and *Pseudochaetosphaeronema* (Ahmed et al., 2015) are capable of causing skin infections in both humans and mammals, such as tinea nigra and subcutaneous phaeohyphomycosis. *Ramichloridium*, *Pseudozyma*, *Rhodotorula*, and *Sporobolomyces* cause hematomycosis (Arendrup et al., 2014; Etienne et al., 2016). *Macrophomina* (Premamalini et al., 2012), *Thermomyces* (Sivagnanam et al., 2013), *Engyodontium alum* (Augustinsky et al., 1990; Thamke et al., 2015; Wu et al., 2016) can induce different types of inflammatory diseases, such as keratitis and endocarditis. *Cladophialophora*, *Chaetomium*, *Curvularia*, *Alternaria*, and *Ramichloridium* are not only pathogens but also cause allergic reactions in humans (McAleer et al., 1981; Cruz et al., 1997; Paredes et al., 2013). More importantly, an increasing number of pathogenic fungi previously considered to be plant pathogens have recently been confirmed to be potential pathogens to humans (Fleming et al., 2002). Most of the newly identified human fungal pathogens are resistant to traditional antifungal drugs. Therefore, a more comprehensive risk assessment strategy for fungal pathogens derived from shrimp is required to effectively protect public health.

## Conclusion

In this study, we used a high-throughput sequencing approach to explore the hepatopancreas and intestinal mycobiota of *L. vannamei*. The results disclosed that (1) the composition of the gut and hepatopancreas of *L. vannamei* is highly diverse and different from that of humans; (2) in winter, the diversity and composition of the mycobiota had a significant difference between the intestinal and hepatopancreas; (3) seasonal variation and diseases considerably affected the fungal microbiota of *L. vannamei*; (4) healthy shrimps was significantly enriched in the genera *Didymella* and *Filobasidium* in the gut, and *Pyrenochaetopsis* in the hepatopancreas. Sick groups had an increased abundance of the genera *Candida* in the intestine. The different fungi may be fungal indicators for health or diseases progression.

## Ethics Statement

All experimental animal protocols in this study were approved by the committee on the Ethics of Animal Experiments of the Guangdong Institute of Applied Biological Resources and followed the basic principles.

## Author Contributions

JC and JL designed the research. JL, HJ, LL, and XZ sampled together. JL, HJ, and LL completed the research. JL, LL, and XZ sorted and analyzed the data. JL and JC wrote and revised the manuscript. All authors approved the final version of the manuscript.

## Conflict of Interest Statement

The authors declare that the research was conducted in the absence of any commercial or financial relationships that could be construed as a potential conflict of interest.

## Footnotes

1 http://unite.ut.ee/index.php

2 http://www.ebi.ac.uk/Tools/msa/clustalw2/

3 http://www.mothur.org/

## Supplementary Material

FIGURE S1 Operational taxonomic units (OTU) number of each group.

FIGURE S2 Rarefaction curves. **(A)** OTU number rarefaction curves for summer samples; **(B)** OTU number rarefaction curves for winter samples; **(C)** Shannon index rarefaction curves for summer samples; **(D)** Shannon index rarefaction curves for winter samples.

FIGURE S3 Species relative abundance curve. Red boxplots depict the sum of species; green boxplots depict the emerging probability of share species.

TABLE S1 The sequencing data for each sample.

TABLE S2 All the obtain OTUs and taxonomy.

TABLE S3 **(A)** Differences in alpha diversity between intestine and hepatopancreas. **(B)** Differences in alpha diversity between summer and winter intestine; and between summer and winter hepatopancreas. **(C)** Differences in alpha diversity between sick and healthy group.
