# Supplementary figures and images for: The Effect of Disease and Season to Hepatopancreas and Intestinal Mycobiota of Litopenaeus vannamei
Source: Front Microbiol. 2019 Apr 24;10:889. doi: 10.3389/fmicb.2019.00889 (PMC6491898; doi:10.3389/fmicb.2019.00889)

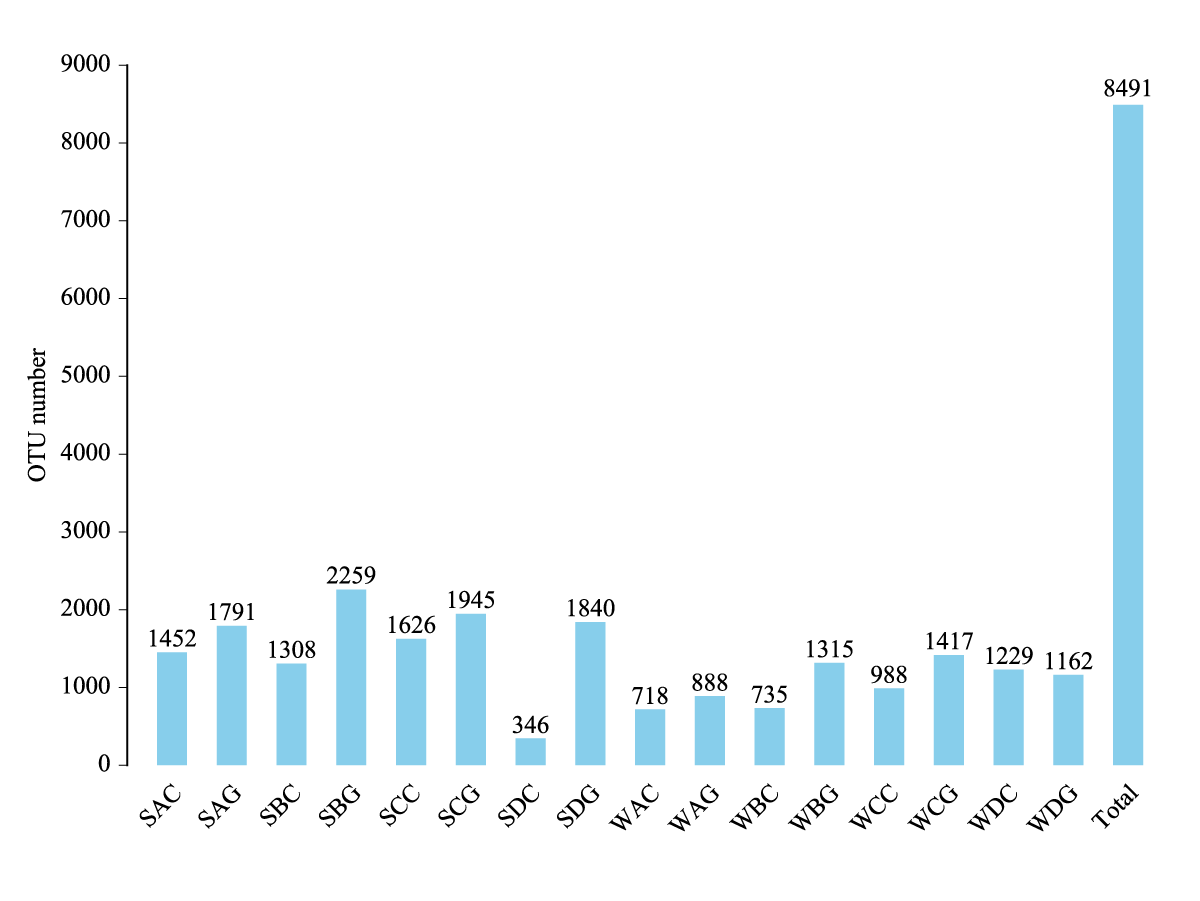

Supplement: FIGURE S1 — Operational taxonomic units (OTU) number of each group. [file Image_1.TIF]

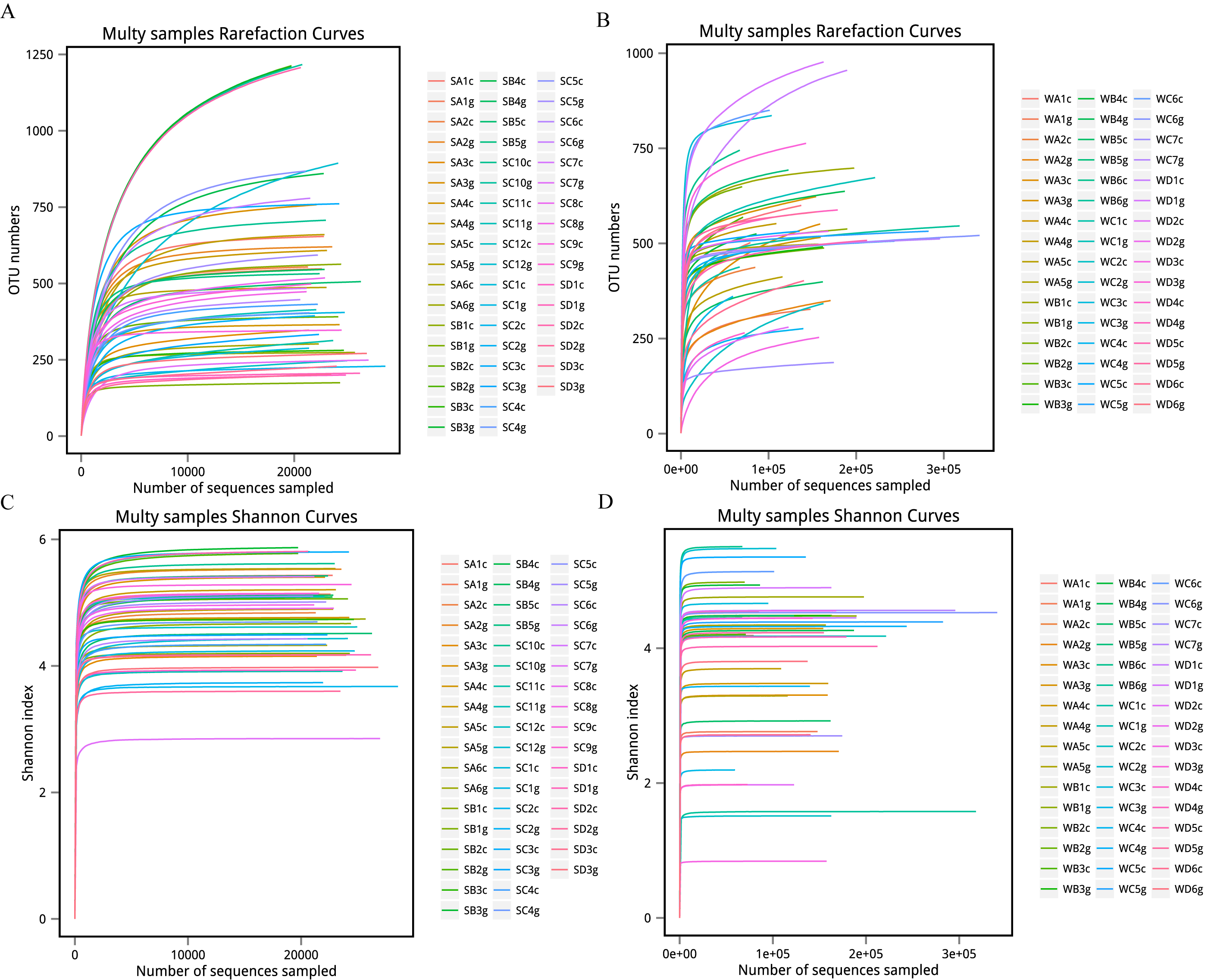

Supplement: FIGURE S2 — Rarefaction curves. (A) OTU number rarefaction curves for summer samples; (B) OTU number rarefaction curves for winter samples; (C) Shannon index rarefaction curves for summer samples; (D) Shannon index rarefaction curves for winter samples. [file Image_2.TIF]

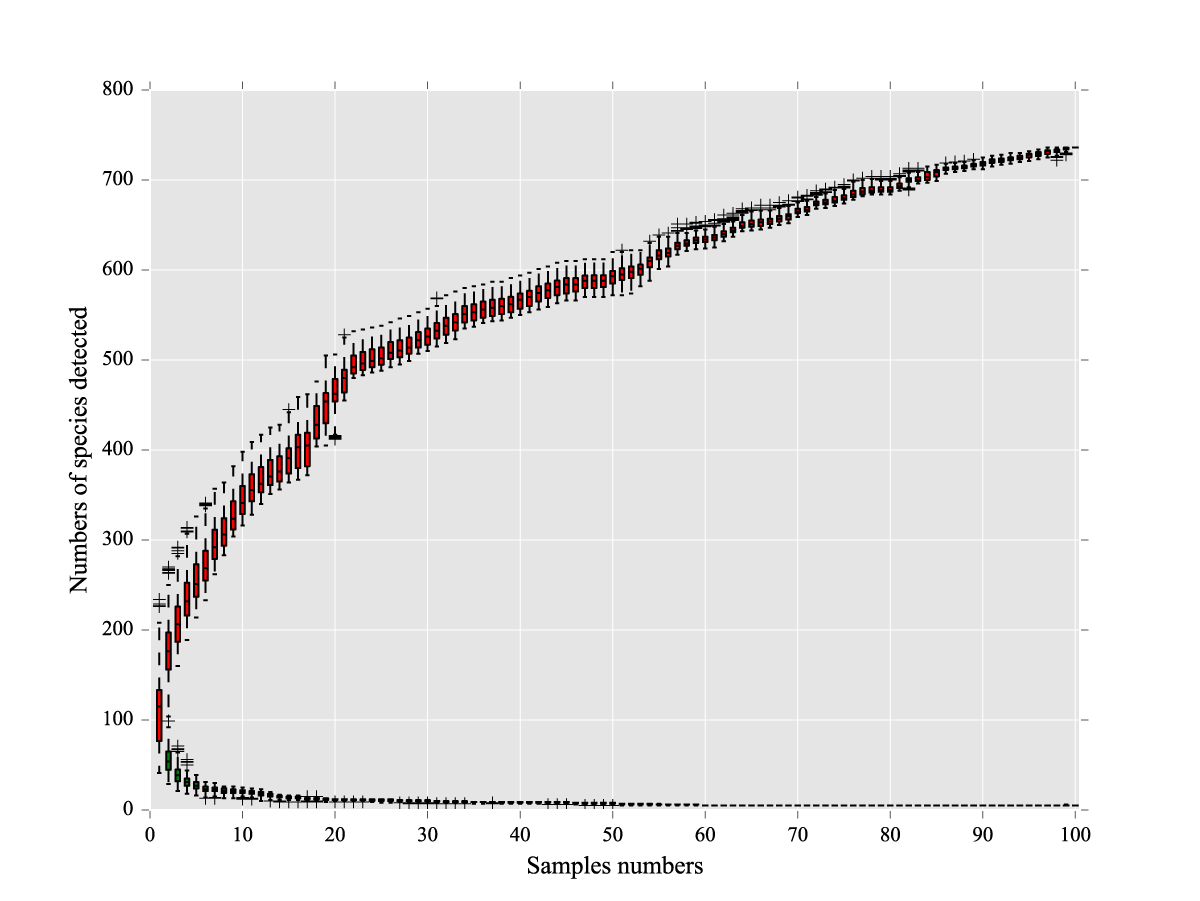

Supplement: FIGURE S3 — Species relative abundance curve. Red boxplots depict the sum of species; green boxplots depict the emerging probability of share species. [file Image_3.TIF]
